# Centella asiatica promotes antioxidant gene expression and mitochondrial oxidative respiration in experimental autoimmune encephalomyelitis

**DOI:** 10.21203/rs.3.rs-3393042/v1

**Published:** 2023-10-06

**Authors:** Payel Kundu, Kanon Yasuhara, Mikah S Brandes, Jonathan A Zweig, Cody J Neff, Sarah Holden, Kat Kessler, Steven Matsumoto, Halina Offner, Carin Stewart Waslo, Arthur Vandenbark, Amala Soumyanath, Larry S Sherman, Jacob Raber, Nora E Gray, Rebbeca Irene Spain

**Affiliations:** Oregon Health & Science University; Oregon Health & Science University; Oregon Health & Science University; Oregon Health & Science University; Oregon Health & Science University; Oregon Health & Science University; Oregon Health & Science University; Oregon Health & Science University; Oregon Health & Science University; Portland VA Medical Center; Oregon Health & Science University; Oregon Health & Science University; Oregon Health & Science University; Oregon Health & Science University; Oregon Health & Science University; Portland VA Medical Center

**Keywords:** Centella asiatica, Antioxidant, Experimental autoimmune encephalomyelitis, Mitochondrial respiration

## Abstract

Centella asiatica (Centella) is a traditional botanical medicine that shows promise in treating dementia based on behavioral alterations seen in animal models of aging and cognitive dysfunction. In order to determine if Centella could similarly improve cognitive function and reduce disease burden in multiple sclerosis (MS), we tested its effects in the neuroinflammatory experimental autoimmune encephalomyelitis (EAE) model of MS. In two independent experiments, C57BL/6J mice were treated following induction of EAE with either a standardized water extract of Centella (CAW) or placebo for 2 weeks. At the dosing schedule and concentrations tested, CAW did not improve behavioral performance, EAE motor disability, or degrees of demyelination. However, CAW-treated mice demonstrated increases in nuclear factor (erythroid-derived 2)-like 2 and other antioxidant response element genes, and increases in mitochondrial respiratory activity. Caw also decreased spinal cord inflammation. Our findings indicate that CAW can increase antioxidant gene expression and mitochondrial respiratory activity in mice with EAE, supporting investigation of the clinical effects of CAW in people with MS.

## 1. Introduction

Cognitive dysfunction is a frequent and disabling symptom in multiple sclerosis (MS), an autoimmune neuroinflammatory disorder of the central nervous system (CNS) that is also characterized by motor and sensory deficits. Information processing speed deficits occur early in the disease course, and are joined over time by difficulties with verbal fluency, verbal episodic memory, visuospatial construction and executive dysfunction [1, 2]. Treatment for MS-related cognitive dysfunction remains elusive. Current MS disease-modifying therapies (DMT), while highly effective at reducing neuroinflammatory attacks, have only a small to moderate positive effect on MS cognition as shown in a meta-analysis of 41 studies and 7, 131 patients [3]. This study further identified no differences in cognitive benefit between lower-efficacy and higher-efficacy DMT, suggesting a pathological basis of MS cognitive dysfunction alternative or additional to neuroinflammation. FDA-approved dementia medications donepezil and memantine failed to reduce cognitive dysfunction in MS trials [4, 5]. The popular supplements gingko biloba and vitamin D similarly failed in clinical trials for that purpose [6, 7]. Cognitive behavioral therapy can improve processing speed dysfunction in people with MS, but suffers from limited access due to factors like proximity to services, health insurance coverage, and time required to participate [8].

Centella asiatica (Centella) is a traditional botanical medicine used to support age-related cognitive health. Preclinical studies using a standardized water extract of Centella (CAW) reveal remarkable cognitive-behavioral enhancing and neuroprotective properties in Alzheimer’s dementia models and aged wild type (WT) animals [9–12]. Other formulations of Centella including a lyophilized powder and the isolated triterpenoid constituent asiaticoside have also proven beneficial in streptozotocin and Aβ1–42 - induced mice, respectively [13, 14]. Proposed mechanisms of CAW in these studies include increases in antioxidant response element (ARE) gene expression and mitochondrial respiration, heightened dendritic arborization, and inhibition of microglial activation.

Clinical trials of Centella suggest that it can lead to improved memory and reduced anxiety [15]. Tiwari et. al. conducted an open-label trial of a 500mg capsules of powdered Centella or placebo twice daily for 6 months in 60 participants over 65 years with mild cognitive impairment. Pre- to post-treatment mini mental state examination (MMSE) scores significantly improved, as did the proportion of participants reporting feelings of well-being and sensory discomfort [16]. A randomized controlled trial of doses ranging from 250mg to 750mg daily of a Centella extract for 2 months in 28 elderly participants resulted in improved working memory, alertness and calmness [17]. An open-label trial of a hydro-ethanolic extract of Centella improved symptoms of generalized anxiety in 33 participants [18]. While these studies are limited by small sample sizes, inadequate descriptions of the investigational products, and in some cases lack of placebo controls, they encourage further exploration of the clinical benefit of Centella.

The pathophysiology of MS suggests it would be amenable to treatment with Centella. In addition to T- and B-cell mediated neuroinflammation leading to demyelination and axonal transection, MS pathophysiology also includes the neurodegenerative pathologies of oxidative stress, mitochondrial dysfunction, and smoldering inflammation that includes chronically activated microglia [19]. MS neurodegeneration is visualized as accelerated CNS atrophy on neuroimaging and characterized clinically with symptoms of cognitive dysfunction and fatigue [20]. We hypothesized that Centella, by treating MS neurodegenerative pathology would benefit cognition and behavior in MS. We therefore determined if benefits to behavioral performance, ARE gene expression, and mitochondrial respiratory activity seen in dementia and aging mouse models would also be observed in an experimental autoimmune encephalomyelitis (EAE) model of MS.

## 2. Materials and Methods

### 2.1. CAW Production, Analysis, and Administration

CAW used in this study was produced as previously described [15]. Briefly Centella asiatica herb (4kg) was extracted by boiling in water (50 L) for 90 minutes. The extract was filtered to remove solid plant debris, the liquid filtrate divided between several aluminum trays and frozen. The frozen extract was lyophilized in 3 separate runs to yield a dry residue (CAW; total weight from 3 batches, 820g). Voucher samples of the starting plant material (BEN-CA-6) and the three dried CAW batches (BEN-CAW-7, 8 and 9) were stored at OHSU’s BENFRA Botanical Dietary Supplements Research Center.

### 2.2 CAW Phytochemical Analysis

Studies by our group and others indicate that triterpene and caffeoylquinic acid components are involved in CAW’s neurotropic, cognitive enhancing, antioxidant activities as well as its ability to improve mitochondrial function [11, 15, 21, 22]. The analysis of triterpenes and caffeoylquinic acids in BEN-CAW-8, using liquid chromatography coupled to multiple reaction monitoring mass spectrometry (LC-MRM-MS), was performed by the BENFRA Botanical Dietary Supplements Research Center at OHSU and has been described in detail [23]. Briefly chromatographic separation was performed using an Intersil Phenyl-3 column, eluting with a 7 minute gradient of methanol in water, both containing 0.1 % formic acid. This system allowed for the resolution of 3 mono-caffeoylquinic acids, 5 di-caffeoylquinic acids and four triterpenes, which were detected using negative electrospray ionization and MRM-MS. The relevant MRM transitions used and % w/w content in BEN-CAW-8 for each of these compounds were as follows: 3-caffeoylquinic acid (353/191; 0.72% ), 4-caffeoylquinic acid (353/173; 0.30%), 5-caffeoylquinic acid (353/191; 0.34%), 1,3-dicaffeoylquinic acid (515/179; 0.25%), 1,5-dicaffeoylquinic acid (515/191; 0.39%), 3,4-dicaffeoylquinic acid (515/173; 0.23%), 3,5-dicaffeoylquinic acid (515/191; 0.20%), 4,5-dicaffeoylquinic acid (515/173; 0.22%), asiatic acid (533; 487; 0.08%), madecassic acid (549/503; 0.14%), asiaticoside (1003/957; 1.46%), madecassoside (1019/973; 3.45%).

### 2.3 CAW administration to EAE mice

For both studies, CAW was dosed at 500 mg/kg/day using 2 drops CAW solution (125mg/ml) dissolved in 5% sucrose/phosphate buffered saline (PBS) solution. Placebo consisted of an equal volume of 5% sucrose/PBS solution. Placebo and CAW solutions were stored at −20°C until use. A fresh vial of placebo or CAW treatment was thawed for use on each day to avoid repeated freeze-thawing then loaded into individual pipette tips for oral administered.

### 2.4. Animals

All procedures were conducted in strict accordance with Federal and NIH Guidelines for the Care and Use of Laboratory Animals and were approved by the Institutional Animal Care and Use Committee of the Portland VA Medical Center (PVAMC) and Oregon Health & Science University (OHSU).

Study 1: 8-12-week old female C57BL/6J mice (n = 20; Jackson Labs, Sacramento, CA, USA) were divided into 2 cohorts, and housed in mixed-treatment groups of 4 per cage. Mice were maintained in the Animal Resource Facility at the PVAMC on a 12-hour light/dark cycle with access to food and water ad libitum. Each cohort was inoculated on a single day with an emulsion containing 200μg Freund’s complete adjuvant/200μg MOG-35-55 peptide with intraperitoneal booster injections of 75ng and 200ng pertussis toxin on Day 2. Starting at EAE clinical disability score 2, mice received daily treatment with either CAW or placebo in a 1:1 distribution. Saline perfusion followed by euthanasia by cervical dislocation occurred after 14 days of treatment on 2 pre-planned days. Half of the mice underwent immediate brain dissection for harvesting of cerebral cortex while the other half underwent brain and spinal cord dissection for subsequent fluorescence-activated cell sorting (FACS).

Study 2: Four-month old female C57BL/6J mice (n = 30; Jackson Labs, Sacramento, CA, USA) were divided into 3 equally-sized mixed treatment groups and matched by initial weight. Mice were house at the OHSU animal facility. Control mice received sham inoculation and 2-day booster with saline followed by placebo treatment (5% sucrose in PBS). The remaining 2 groups received EAE induction as described above followed by either placebo treatment (PBO) or CAW (dosed as in Study 1) assigned 1:1 based on weight. Treatments began on Day 6 after inoculation each morning and continued for 14 days including the day of euthanasia (Day 20). Several animals required supplemental saline treatments due to weight loss. Following euthanasia which was performed by cervical dislocation, all mice immediately underwent brain and spinal cord dissection with half of all brains harvested for cerebral cortex while lumbar spinal cords were prepared for immunohistochemistry (IHC).

### 2.5. Weight, EAE disability and behavioral testing

Weight was recorded at baseline and daily in Study 1 and at baseline and starting Day 5 (pretreatment) in Study 2. EAE clinical disability was scored daily by the same technician using a scale from 0 (no disability) to 6 (moribund requiring euthanasia). A score of 2 indicates moderate hindlimb weakness or mild ataxia. A final cumulative disease index (CDI) score was calculated as the sum of the daily scores over the treatment period as previously described [24].

Behavioral performance including exploratory activity and anxiety measures were assessed in the open field test as previously described [25]. The open field consisted of a well-lit square (L 40.6 × W 40.6 × H 40.6 cm) with a central light intensity of 100 lx. Mice were allowed to explore the open field for 10 minutes during two consecutive days. Two adjacent arenas were used simultaneously for testing. The enclosures were cleaned with 0.5% acetic acid between trials. Performance of mice was tracked using Ethovision 15 XT software. Performance measures analyzed were total Distance Moved (general activity), Center Duration (anxiety), Latency to the First Center Entry, and Center Frequency (Study 2 only). Open field testing occurred on the final study day for Study 1 and on Days 5 (pretreatment), 12 (early disability), and final Day 20 in Study 2. Cognitive tests were not conducted due to the short treatment duration limiting the assessment window, and concern for confounding of test results caused by EAE motor weakness.

### 2.6. Fluorescence Activated Cell Sorting (FACS) and Immunohistochemistry (IHC)

Inflammation including microglial activation was evaluated from dissected brain and spinal cords in Study 1 via FACS analyses. Leukocytes were labelled for mononuclear cells (CD74+) and microglia (CD45loCD11b + and CD45hiCD11b+) using monoclonal antibodies as previously described [26]. Due to expected low cell counts, tissues from animals within treatment groups were pooled prior to FACS testing.

For IHC and tissue staining, tissues were fixed in 4% paraformaldehyde, freeze-embedded, and serially-sectioned at a thickness of 10μm on a cryostat (Leica, Wetzler, Germany) and placed on glass slides. Tissues were then either immunostained with anti-CD3 antibodies (BD Pharmigen, 1:300; to label inflammation) or anti-Iba1 antibodies (Wako, 1:300; to label activated microglia) along with Hoechst 3342 (to label cell nuclei), followed by detection with fluorescence-labeled secondary antibodies as previously described [27]. Myelin was detected using fluoromyelin (ThermoFisher Scientific) as previously described [28].

### 2.7. ARE gene expression and mitochondrial respiratory activity

Gene expression and mitochondrial respiratory activity were quantified in cortical synaptosomes. Synaptosomes were freshly isolated from one half of the cortical tissue from the left hemisphere using Syn-per reagent (Thermo Scientific #87793) as per the manufacturer’s protocol. Total RNA was collected via Tri-Reagent extraction (Molecular Research Center, Cincinnati, OH, USA) and cDNA produced via reverse transcription using the Superscript III First Strand Synthesis Kit (Invitrogen, Carlsbad, CA, USA) per manufacturers’ instructions. Relative gene expression was measured using Taqman primers and probes (nuclear factor (erythroid-derived 2)-like 2 (Nfe2l2; Nrf2 - Mm00477784_m1), NAD(P)H dehydrogenase-quinone oxidoreductase 1 (Nqo1; Mm001253561_m1), glutamate-cysteine ligase, catalytic subunit (Gclc; Mm00802655_m1), heme oxygenase 1 (HMOX1, Ho-1; Mm00516005_m1) and reagents (TaqMan Gene Expression Master Mix) from Applied Biosystems (Foster City, CA, USA) with normalization to glyceraldehyde-3-phosphate dehydrogenase (Gapdh; hs02758991_g1 (Applied Biosystems, Foster City, CA, USA)) expression. qPCR was performed on a StepOne Plus Machine (Applied Biosystems, Foster City, CA, USA) and analyzed using the delta-delta Ct method. All groups were normalized to control mice.

Mitochondrial bioenergetics were assessed in cortical synaptosomes using the Seahorse XFe96 Analyzer. The total protein concentration of the synaptosomal preparation for each mouse was determined using a bicinchoninic acid (BCA) assay. A total of 10ug of synaptosomal protein diluted in 25uL mitochondrial assay solution (MAS; 70mM sucrose, 220mM mannitol, 10mM KH2PO4, 5mM MgCl2, 2mM HEPES, 1 mM EGTA, 0.2% BSA) was plated in each well of a polyethylenimine (PEI) coated 96 well Seahorse plate (5–6 replicate wells for each animal) and the plate was centrifuged at 1200g for 1h at 4C. 155 μL of Agilent Seahorse XF DMEM Medium pH 7.4 (Part # 103575-100) supplemented with 1 mM pyruvate, 2 mM glutamine, and 10 mM glucose was plated in each well of the 96 well plate.

Mitochondrial function was then assessed using the MitoStress kit (Agilent #103015-100) as previously described [29]. Briefly, oxygen consumption rate (OCR) was measured under varying conditions. After three initial baseline measurements of OCR, the adenosine triphosphate (ATP) synthase inhibitor oligomycin (2μM) was added and three subsequent measurements were taken. Next an electron transport chain accelerator, p-trifluoromethoxy carbonyl cyanide phenyl hydrazone (FCCP) at 2 μM was added and 3 measurements of maximal respiration were taken. Finally, the mitochondrial inhibitors rotenone (0.5μM) and antimycin (0.5μM) were added and three final measurements were taken. Spare capacity was calculated by subtracting the average of the basal measurements from the average of the three maximal measurements following FCCP administration.

### 2.8. Statistical Analyses

Study 1: Mean change in weights, CDI scores, behavioral tests, and ARE gene expression were compared between groups by t-test at study end. For the Latency to First Center entry, animals that never entered the center were given latency of the full trial length (600 seconds). Mitochondrial function was compared using repeated measures ANOVA across the study profile and by t-tests between groups at basal, maximal, and spare capacity endpoints. Because FACS analyses used pooled tissues (3 mice per treatment group contributed to each sample tested) due to expected low CNS cell numbers, statistical error could not be calculated; Instead, results were qualitatively compared.

Study 2: Mean change in weights were compared between groups by repeated measures ANOVA with Tukey’s post hoc analysis were employed for group differences in daily weights starting Day 5 and cognitive behavioral scores at Days 12, and 20. CDI were compared by t-test between CAW- and placebo-treated mice. ARE gene expression groups were compared using ANOVA with Dunnett’s post-hoc tests for pairwise comparison. For mitochondrial respiration, repeated measures ANOVA was calculated across the profile and a separate ANOVA was done for each endpoint (i.e. one for basal respiration, another for maximal respiration, etc.) with Tukey’s post-hoc for pairwise comparison. IHC results for CD3 and Iba1 counts were analyzed with T-tests between placebo and CAW- treated EAE mice. Significance for all tests was set at p < 0.05.

## 3. Results

### 3.1. Safety and tolerability

Of the 20 mice in Study 1, only 18 received treatment with CAW or placebo (n = 9 per group) as 2 animals did not reach a clinical disability score of 2 in sufficient time to receive treatment. Because of the pre-planned sacrifice dates, there were intervals ranging from 1 to 5 days between the 14th day of treatment and sacrifice. One placebo-treated mouse received 12 days of treatment instead of 14. All 30 mice in Study 2 received 14 days of treatment up to and including on the day of sacrifice. There was no difference in mean weight change between the treatment groups in Study 1 (0.66g SEM 0.44 vs 0.28g SEM 0.38, p = 0.26). While there was an effect of group on body weight in Study 2 (F[2, 27] = 3.350, p = 0.050; Tukey’s post hoc Control vs CAW: p = 0.040), there was no difference in the changes in weight between CAW and placebo cohorts.

### 3.2 Clinical disability, inflammation, and demyelination

**Figure 1:**
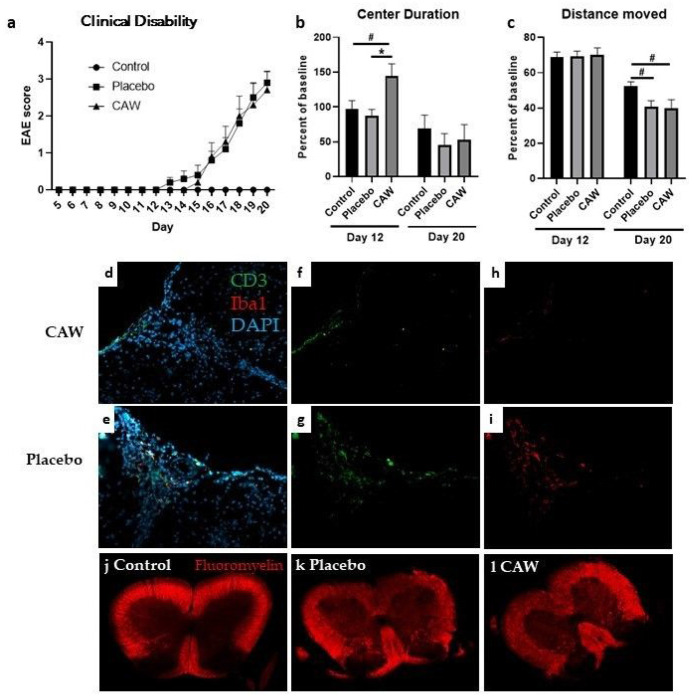
a. Onset and final clinical disability in CAW-treated mice and placebo-treated mice following induction of experimental autoimmune encephalopathy (EAE) were similar between groups in Study 2. Control mice without EAE did not exhibit clinical disability b. Center Duration as a percent of baseline was greater in CAW-treated mice (less anxiety) than placebo or control cohorts at day 12 (early symptomatic) but not at Study 2 end. c. Distance Moved (activity) at Study 2 day 20 was lower in both CAW and placebo group compared to controls, likely reflecting disability caused by EAE. d., e. Composite stains for CD3 (green, lymphocytes) and Iba1 (red, activated microglia) are shown with DAPI (blue, DNA) in a CAW and placebo-treated mouse. f., g. Decreased CD3 staining for in a CAW-treated mouse compared to placebo. h., i. A trend toward decreased Iba1 staining for activated microglia between CAW and placebo-treated mice. j., k., l. While both EAE spinal cord sections demonstrate demyelination compared to control, there were no qualitative difference between placebo and CAW

Mean CDI did not differ between CAW and Placebo groups in either study. While the onset of mean clinical disability in CAW-treated mice in Study 2 (Day 15) occurred 2 days after the mean onset in placebo-treated mice (Day 13), disability equalized by Day 16 and remained similar in both cohorts to study end (Fig. 1a).

Qualitatively, FACS analysis of pooled spinal cord samples of the 3 CAW-treated mice in Study 1 demonstrated decreased monocyte (CD74+) expression compared to the 3 placebo-treated mice (15% v 19.7%) but had similar expression in the pooled brain samples. Conversely, a more favorable activated (CD11bCD45hi) to resting (CD11bCD45lo) microglial profile was noted in CAW-treated brains (16.7%/58.3%) vs Placebo (24.9%/54.1%) but not in the CAW-treated spinal cords (51.5%/19.0%) vs placebo (46.6%/17.7%).

IHC of lumbar spinal cords of the 9 CAW-treated mice in Study 2 revealed decreased CD3 counts normalized to total cell counts compared to the 10 placebo-treated mice (2.9% STD 1.6% vs 5.0% STD 1.7%, p = 0.01, Fig. 1: f&g). Normalized Iba1 counts in the lumbar spinal cords among the 10 CAW-treated mice demonstrated a trend towards reduced microglial activation but was not significantly different from the 9 placebo mice (3.3% STD 2.8% vs 5.2% STD 5.1%, p = 0.30, Fig. 1: h&i). There was no qualitative difference in myelination between mice treated with CAW or placebo (Fig. 1: k&l).

### 3.3 Behavioral performance

Overall, CAW did not impact behavioral performance in Study 1 including Distance Moved, Center Duration, and Latency to First Center Entry. In Study 2, the Center Duration times on day 12 (early symptomatic) were longer in the CAW-treated mice (F[2, 27] = 5.229, p = 0.012; Tukey’s post-hoc: p = 0.014 CAW vs placebo, p = 0.050 CAW vs control) a finding that was not seen at study end (Fig. 1b). There was an effect of group on Distance Moved at study end (F[2, 27] = 3.433, p = 0.047), however this was driven by EAE and not by CAW treatment (Fig. 1c). Tukey’s post-hoc pairwise comparisons did not reach significance for control vs CAW (p = 0.064) or control vs placebo (p = 0.097). There were no effects of group for the Latency to First Center Entry or for Center Frequency.

### 3.4 ARE gene expression and mitochondrial respiratory activity

**Figure 2:**
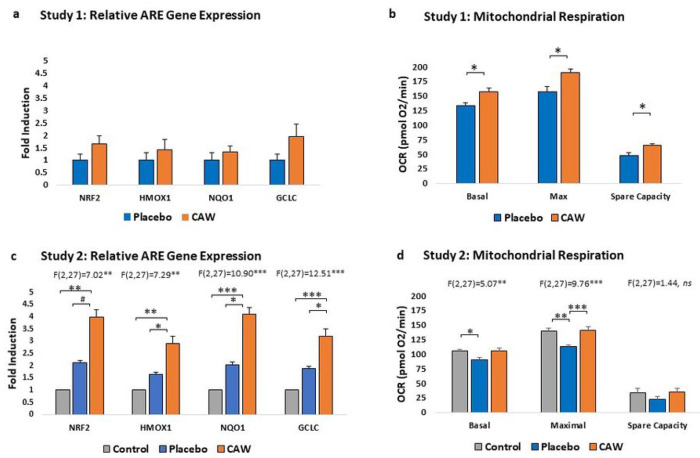
a. Study 1: Greater, but non-significant, induction of antioxidant response element (ARE) gene expression in cerebral cortices of CAW-treated mice than placebo (n=4 per group). b. Mitochondrial respiration measured as oxygen consumption rates (OCR) of CAW-treated mice were significantly greater than placebo at basal, maximal, and spare capacity endpoints in Study 1. c. Study 2: ARE gene expression was significantly greater in CAW- compared to placebo-treated mice. Greater ARE gene induction in placebo than control mice reflect the compensatory antioxidant response to experimental autoimmune encephalomyelitis. d. Study 2 additionally demonstrated normalization of mitochondrial respiration in CAW mice to those of controls without EAE. Pairwise differences in OCR between CAW and placebo in Study 2 reached significance at the maximal endpoint. HMOX1, heme oxygenase 1; GCLC, glutamate-cysteine ligase, catalytic subunit; NQ01, NAD(P)H dehydrogenase-quinone oxidoreductase 1; NRF2, nuclear factor (erythroid-derived 2)-like 2. *p<0.05, **p<0.01, ***p<0.001, #p<0.10

CAW-treated mice demonstrated a non-significant trend towards increased ARE gene expression in cerebral cortex over placebo-treated mice (n = 4 per group) in Study 1 (Fig. 2a, Table 1). However, in Study 2, ARE gene expression was significantly impacted by treatment group, with CAW-treated gene expression elevated compared to both placebo-treated EAE animals and healthy controls (n = 10 per group, Fig. 2b). Notably, gene expression in placebo animals was greater than in control animals reflecting the expected increase in ARE gene expression as a response to EAE.

CAW-treated mice also demonstrated significantly greater cortical mitochondrial respiratory activity than placebo-treated animals in Study 1 across the study profile (F(1,2) = 42.64, p < 0.001) and between groups at each study endpoint (Fig. 2b, Table 1). Mitochondrial respiratory activity was also significantly influenced by group in Study 2 (F[2, 27] = 3.601, p = 0.041), with Tukey’s post-hoc for CAW vs placebo reaching significance (p = 0.0005, Fig. 2d). Oxygen consumption rates in CAW-treated animals were restored to those of healthy control mice.

## 4. Discussion

In two independent studies, we found that treatment with CAW, a water extract of the botanical Centella asiatica, significantly increased cortical ARE gene expression and mitochondrial respiratory activity, and reduced spinal cord inflammation in mice induced with EAE. The effects of CAW specifically on microglial activation were equivocal, and, besides reduced measures of anxiety on day 12, there were no effects on behavioral performance, on clinical disability, or on demyelination.

The increases in ARE gene expression and mitochondrial respiratory activity in our studies are consistent with those found in studies using dementia and aging mouse models. CAW-induced increases in ARE gene expression were observed in the frontal cortex and hippocampi of 5xFAD Aβ-amyloid mice, and in 18-month (old) C57BL/6J mice [12]. Interestingly, while young (6-week-old) C57BL/6J mice treated with CAW also responded to CAW with increases in ARE gene expression, cognitive improvements were primarily observed in the old mice [10}. Mitochondrial respiratory activity improved in 5xFAD Aβ-amyloid mice in response to CAW treatment but there was no change in mitochondrial respiration in WT mice after CAW treatment suggesting a ceiling effect for this biomarker [12]. Indeed, CAW-treated EAE mice in our Study 2 demonstrated restoration of mitochondrial respiration that met, but did not exceed, the bioenergetic profile of control mice.

Conclusions regarding the effects of CAW treatment on neuroinflammation and microglial activation based on our study are limited due to the different evaluation methods used in Studies 1 and 2, and other factors. Mitochondrial dysfunction and oxidative stress are postulated to be both a cause and result of inappropriate microglial activation in MS, although tissue damage caused by EAE inflammation limits evaluation of these pathologies [30]. Newer models may circumvent this issue and could be considered for future evaluations of CAW in EAE [31].

The lack of behavioral changes, aside from reduced measures of anxiety on day 12, in response to CAW treatment in our studies has several possible explanations. While mice induced with EAE are reported to display behavioral alterations, a common concern is that the motor disability from EAE obstructs the assessment of behavioral performance [32]. However, given that aside from one measure, behavioral performance did not differ between early symptomatic testing and final testing, motor weakness was less likely to have confounded our results. Other possible explanations are that the relatively young mice in our studies may not have manifested behavioral changes from EAE, that the behavioral tests chosen were not sensitive to EAE-induced injury, that the short treatment duration precluded detection of behavioral benefits, and that either the CAW dose or the number of mice treated were insufficient to detect treatment responses. Finally, it’s possible that CAW simply does not affect behavioral performance in EAE but may have other not yet tested clinical correlates to the improved biomarkers.

Limitations of this study were the different designs of Studies 1 and 2 preventing combining data for analyses, and in particular the differences in treatment durations prior to sacrifice. Cognitive testes were not assessed in this study for reasons previously discussed which precludes conclusions about CAW effects on EAE cognition and prevents comparison with studies in other disease model results that did conduct cognitive tasks. Other limitations of behavioral testing and evaluation of neuroinflammation were discussed previously.

In conclusion, the EAE model of MS repeats the CAW-induced increases in ARE gene expression and mitochondrial respiratory activity seen in aging and dementia models. Further exploration will determine the clinical correlates to the striking improvements in these potential MS biomarkers.

## Figures and Tables

**Table 1:** Antioxidant response element (ARE) gene expression induction and mitochondrial respiratory activity as measured by oxygen consumption rate (OCR) were compared in Study 1 between CAW-treated and placebo-treated mice with experimental autoimmune encephalomyelitis by t-tests. For Study 2, Dunnett’s pairwise comparisons for ARE gene expression and Tukey’s post hoc for OCR are presented below. ANOVA results are presented in the text. Sample sizes were n = 9 per group for Study 1 and n = 10 per group for Study 2 with exceptions individually noted.

	Study 1		Study 2		
Outcome	Placebo	CAW	Placebo	CAW	Control
ARE: NRF2 mean fold induction, SEM	1, 0.0.24	1.66, 0.31 *p = 0.15*	2.12, 0.43 *vs control, p = 0.34*	3.99, 0.90 *vs placebo, p = 0.07*	1, 0.23 *vs CAW*, **p = 0.002**
ARE: HMOX1 Mean fold induction, SEM	1, 0.30	1.42, 0.39 *p = 0.43*	1.63, 0.24 *vs control, p = 0.42*	2.90, 0.58 *vs placebo*, **p = 0.047**	1, 0.14 *vs CAW*, **p = 0.002**
ARE: NQO1 Mean fold induction, SEM	1, 0.29	1.31, 0.26 *p = 0.46*	2.03, 0.35 *vs control, p = 0.27*	4.09, 0.71 *vs placebo*, **p = 0.013**	1, 0.29 *vs CAW*, **p = 0.0003**
ARE: GCLC Mean fold induction, SEM	1, 0.24	1.95, 0.48 *p = 0.13*	1.88, 0.26 *vs control, p = 0.12*	3.20, 0.46 *vs placebo*, **p = 0.016**	1, 0.17 *vs CAW*, **p = 0.0001**
OCR -basal (pmol O2/min) mean SEM	133.1, 4.4 n = 3	156.8, 6.4 n = 4, **p = 0.04**	90.08, 3.55 *vs control*, **p = 0.03**	105.56, 4.98 *vs placebo, p = 0.18*	105.20, 2.99 *vs CAW, p = 0.99*
OCR -maximal (pmol O2/min) mean SEM	156.4, 9.2 n = 3	190.28, 5.7 n = 4, **p = 0.03**	112.60, 3.10 *vs control*, **p = 0.001**	140.82, 6.33 *vs placebo*, **p = 0.0005**	138.90, 5.16 *vs CAW, p = 0.96*
OCR- spare capacity (pmol O2/min), mean SEM	47.8, 5.0 n = 3	64.8, 2.8 n = 4, **p = 0.03**	22.52, 4.87 *vs control, p = 0.38*	35.26, 5.29 *vs placebo, p = 0.28*	33.62, 6.97 *vs CAW, p = 0.98*

GCLC, glutamate-cysteine ligase, catalytic subunit; HMOX1, heme oxygenase 1; NQO1, NAD(P)H dehydrogenase-quinone oxidoreductase 1; NRF2, nuclear factor (erythroid-derived 2)-like; SEM, standard error of the mean

## Data Availability

Data is available upon request to the corresponding author.
